# Alzheimer’s disease modeling and drug screening through hiPSC 3D bioprinting

**DOI:** 10.1002/alz.092690

**Published:** 2025-01-03

**Authors:** Stefano Sorrentino, Stefan Wendt, Wenji Cai, Christopher Lee, Declan Brennan, Xiujuan Wu, Haakon B. Nygaard

**Affiliations:** ^1^ 2215 Wesbrook Mall, Vancouver, BC Canada; ^2^ University of British Columbia, Vancouver, BC Canada; ^3^ UBC Hospital Clinic for Alzheimer Disease and Related Disorders, Vancouver, BC Canada

## Abstract

**Background:**

Our current understanding of the molecular mechanisms underlying amyloidogenesis in Alzheimer’s Disease (AD) is limited by the lack of comprehensive models closely resembling human pathology. Human induced pluripotent stem cell (hiPSC) 3‐dimensional (3D) models, such as brain organoids and neurospheres, are emerging as innovative approaches to model neurodegenerative diseases *in vitro*. However, they rely on hiPSC self‐organization and are therefore characterized by low reproducibility and homogeneity. Moreover, they lack proper extracellular matrix (ECM) and microglial cells which both play a pivotal role in amyloid beta (Aβ) plaque dynamics. 3D‐bioprinting is an innovative bioengineering technique that combines biomaterials and live cells, in so‐called ‘bioinks’, to shape, in a layer‐by‐layer fashion, highly geometrical controlled 3D structures. The surrounding ECM improves the exchange of nutrients, oxygen, and drugs making them closer to the physiological fluidic dynamic.

**Method:**

Here, we generated a 3D‐Bioprinted brain model suitable for long‐lasting culturing of iPSCs‐derived cortical neurons and astrocytes starting from neuronal precursor cells (NPCs).

**Result:**

NPCs can be successfully bioprinted in a multilayer wood‐pile structure to mimic the human cerebral cortex architecture with high spatial resolution, low pressure, and high speed maintaining cell viability and proliferation. NPCs can be efficiently expanded and differentiated into functional cortical neurons/astrocytes in 3D cultures. Moreover, when exogenous synthetic Aβ42 is administrated to the culturing medium, hiPSC‐derived microglia can efficiently infiltrate into 3D bioprinted constructs and phagocyte amyloid deposits.

**Conclusion:**

We believe our model will serve to elucidate the early stages of AD by offering a novel perspective that more closely resembles the human AD brain. Furthermore, by using synthetic Aβ enriched bioink and familial AD cell lines that overexpress Aβ our system might offer the chance to study the nucleation of Aβ deposits in real time and mimic the formation of proper Aβ plaques *in vitro* without the involvement of animal models.

